# Enabling earlier detection of spinal lesions in CT imaging with artificial intelligence—a case study

**DOI:** 10.3389/frai.2026.1767814

**Published:** 2026-05-13

**Authors:** Marlene Fritzsche, Patrick Kara-Schmidt, Matthias Kirchler, Kenneth Schroeder, Christian Wiedemeyer, Lea-Elena Braunschneider

**Affiliations:** Floy GmbH, Munich, Germany

**Keywords:** artificial intelligence, clinical decision making, early cancer detection, ML, precision oncology, second reader, spinal metastases

## Abstract

This study investigates whether an artificial intelligence–based second reader can detect malignant spinal lesions on computed tomography earlier than radiologists, thereby supporting precision oncology through more timely diagnosis and treatment planning. The spine is one of the most common locations for metastases in advanced cancer, significantly influencing symptoms, staging and therapeutic decisions. Delayed or missed detection can, however, impair outcomes. A three-dimensional nnU-Net segmentation model trained on 653 scans was applied to a retrospective cohort of 200 patients who later received confirmed diagnoses of malignant spinal lesions; earlier examinations without documented disease were re-evaluated by one board-certified radiologist both unaided and with AI support. The primary measure was the proportion of malignant spinal lesions detected by the model before their baseline reporting, with AI-derived lead time as a secondary endpoint. The system identified 12 malignant spinal lesions that remained invisible to radiologists on unaided retrospective review, achieving a mean lead time of 228 days, and highlighted 25 additional malignant spinal lesions that were retrospectively visible but initially unreported. Across the cohort, AI flagged earlier findings in 37 patients. These preliminary results suggest that AI-assisted CT interpretation may have the potential to identify sub-visual or otherwise overlooked malignant spinal lesions at an earlier scan date than standard radiologist reporting. Further prospective studies are needed to determine whether these findings translate into clinical benefits such as improved diagnostic completeness, earlier treatment initiation, and better patient outcomes.

## 1 Introduction

Early detection of malignant spinal lesions is crucial for improving both the prognosis and quality of life of cancer patients. Timely identification allows intervention that can prevent or reduce skeletal-related events (SREs) such as pathologic fractures, spinal cord compression, and severe bone pain, all of which significantly impair quality of life and can worsen overall prognosis but also influences therapeutic decision-making, while directly influencing therapeutic decision-making within contemporary precision oncology frameworks (Heindel et al., 2014; Ryan et al., 2020; Tanko et al., 2006). Bone tissue is the third most common site for metastases after lungs and liver, particularly in solid tumors like breast, prostate, lung, thyroid, and kidney cancers. The vertebral bodies and pelvis are frequent sites of bone metastasis, due to their high content of red bone marrow and blood flow, which facilitates the seeding of circulating tumor cells (Fornetti et al., 2018). Their presence often signals advanced disease and a poor prognosis. While bone metastases are rarely curable, they can be effectively managed through a multidisciplinary approach involving systemic therapy, bone-targeted agents, and local interventions (Fornetti et al., 2018). Treatment can preserve function, improve quality of life, and, in some cases, extend survival (Ryan et al., 2020). In clinical practice, the detection and characterization of individual, solitary bone lesions are challenging because these lesions can represent both benign and malignant processes. This diagnostic challenge arises because solitary bone lesions encompass a wide spectrum of entities, including benign developmental abnormalities, true neoplasms, and malignant tumors, as well as nonneoplastic conditions that can mimic neoplasms radiographically and histologically (Ahlawat et al., 2025; Kwee et al., 2017; Remotti and Feldman 2012). The American College of Radiology notes that primary bone tumors are conventionally classified as benign, intermediate, or malignant, and that many benign lesions are asymptomatic and indistinguishable from malignant ones based solely on imaging or clinical presentation (Ahlawat et al., 2025). Modern computed tomography (CT) provides high-resolution, cross-sectional imaging of the skeletal system, eliminating the superimposition of structures seen in plain radiography. It enables precise visualization of bone architecture and is highly effective in reliably detecting both osteolytic and osteoblastic (sclerotic) lesions (Heindel et al., 2014). Nevertheless, the sensitivity of radiologists remains limited. Large-scale observational studies have shown that radiologists often overlook subtle or unexpected malignant spinal lesions on CT scans. For instance, according to Onoue et al. (2021) the mean lesion-based sensitivity of radiologists for detecting malignant spinal lesions in serial CT scans was only 33.9%. Even with advanced temporal subtraction methods, detection performance remained low. Similarly, Zhang et al. (2025) reported a lesion sensitivity of only 40.5%, with smaller or unexpected malignant spinal lesions often not being detected, especially under real working conditions with high diagnostic volumes (Onoue et al., 2021). These findings underscore persistent limitations in human image interpretation, particularly in high-volume clinical environments where timely and accurate detection is essential for guiding personalised cancer care.

The integration of artificial intelligence (AI), particularly deep learning models such as nnU-Nets, into radiology workflows shows strong potential to address the limitations of human interpretation in detecting malignant spinal lesions on CT scans. Recent evidence shows that AI-assisted systems can significantly improve the sensitivity and specificity of lesion detection, especially for subtle, osteoblastic, or anatomically complex lesions (Tao et al., 2025). They appear to be particularly effective in supporting less experienced readers. In addition to increased accuracy, deep learning-based tools have been shown to reduce interpretation times and streamline clinical workflows. Within this context, AI-enabled CT interpretation represents a meaningful advancement toward more consistent, reproducible, and timely oncologic assessment as key components of precision oncology (Hoshiai et al., 2022; Noguchi et al., 2022). Our model builds on this foundation by using deep-learning based segmentation algorithms to detect density changes and structural abnormalities in bone tissue.

In this study, we retrospectively reviewed a cohort of patients with reported malignant spinal lesions identified on CT scans and examined their earlier imaging in which no lesion had been diagnosed at the time of initial radiological assessment. Our AI model was applied to these prior scans in a simulated second-reader scenario to evaluate whether the lesions could have been detected earlier with AI support. The objective was to evaluate the contribution of AI-assisted CT analysis to earlier detection of malignant spinal lesions, particularly subtle or unexpected findings that may be overlooked in routine clinical workflows, such as in cases where imaging was performed for unrelated clinical indications, thereby aligning earlier diagnostic insight with the principles of precision oncology.

## 2 Materials and methods

### 2.1 Participants

We retrospectively reviewed patients with radiology reports indicating malignant spinal lesions, dated between April 2020 and August 2024. Inclusion criteria required a baseline radiology report mentioning malignant spinal lesions and at least one earlier CT with a corresponding report that did not mention malignant spinal lesions. Patients meeting these criteria were included in the analysis, resulting in 265 cases. In the first selection stage, the AI model was retrospectively applied to the earlier CT scans of all 265 patients, simulating a second reader scenario. Of these, 65 cases were excluded at this stage because the AI model did not generate a prediction on the prior scan, leaving 200 cases in which the AI had flagged a potential malignant spinal lesion. These 200 cases were taken forward for radiologist evaluation. The Ethics Committee of the Bavarian State Chamber of Physicians approved that according to § 15 of the professional code of physicians of Bavaria retrospective reviews of anonymized patient data do not require ethical approval, and the requirement for a written informed consent from study participants was waived (Reference-No. 2025-1001).

CT scans were performed across 25 outpatient radiology locations throughout Germany, spanning large urban centres with populations of approximately one million to small towns of around 10,000 inhabitants. The participating sites include general radiology practices and hospital departments serving a clinically diverse patient population encompassing both oncology and non-oncology cases.

Lesions were distributed across all spinal regions from C1 to L5, involving the cervical, thoracic, and lumbar spine, with both osteolytic and osteoblastic patterns represented. In some cases, associated extraosseous extension into adjacent soft tissues was present. Case inclusion required precise lesion-level spatial concordance: each AI-flagged finding on the pre-baseline scan had to correspond to the exact same vertebra and location as the lesion subsequently reported at baseline, confirming it was the same lesion regardless of vertebral level (see Image Reading Protocol). On pre-baseline scans, the AI typically identified solitary lesions, consistent with earlier-stage, lower-burden disease; baseline scans more often showed multifocal or extensive disease, reflecting progression in the interval between the two examinations.

### 2.2 Image Reading Protocol and Case Selection

One board-certified radiologist with 7 years of experience performed a two-pass retrospective review of all pre-baseline CT examinations. In the first pass (unaided reading), the radiologist reviewed each scan without access to AI outputs and recorded their assessment of any findings. The second pass (the AI-augmented read) was conducted immediately thereafter for each case. For this, the same radiologist reviewed the AI predictions and for every AI-flagged finding, determined: (1) whether the finding corresponded spatially to the malignant spinal lesion documented in the clinical baseline report; (2) whether the lesion was already visible at the earlier time point; and (3) whether the finding represented malignancy based on the overall clinical course. These assessments were then compared to the unaided read from the first pass. Malignancy status was confirmed retrospectively based on the radiologist’s assessment at the baseline examination and, when available, on subsequent disease progression documented in follow-up imaging and clinical reports. In five of the 12 early detected malignant spinal lesions and ten of the 25 retrospectively visible but unreported malignant spinal lesions, no follow-up beyond the baseline examination was available; in these instances, malignancy determination relied on the baseline report together with the reviewing radiologist’s adjudication. In all cases with follow-up, malignant nature was confirmed over time.

**Figure 1:**
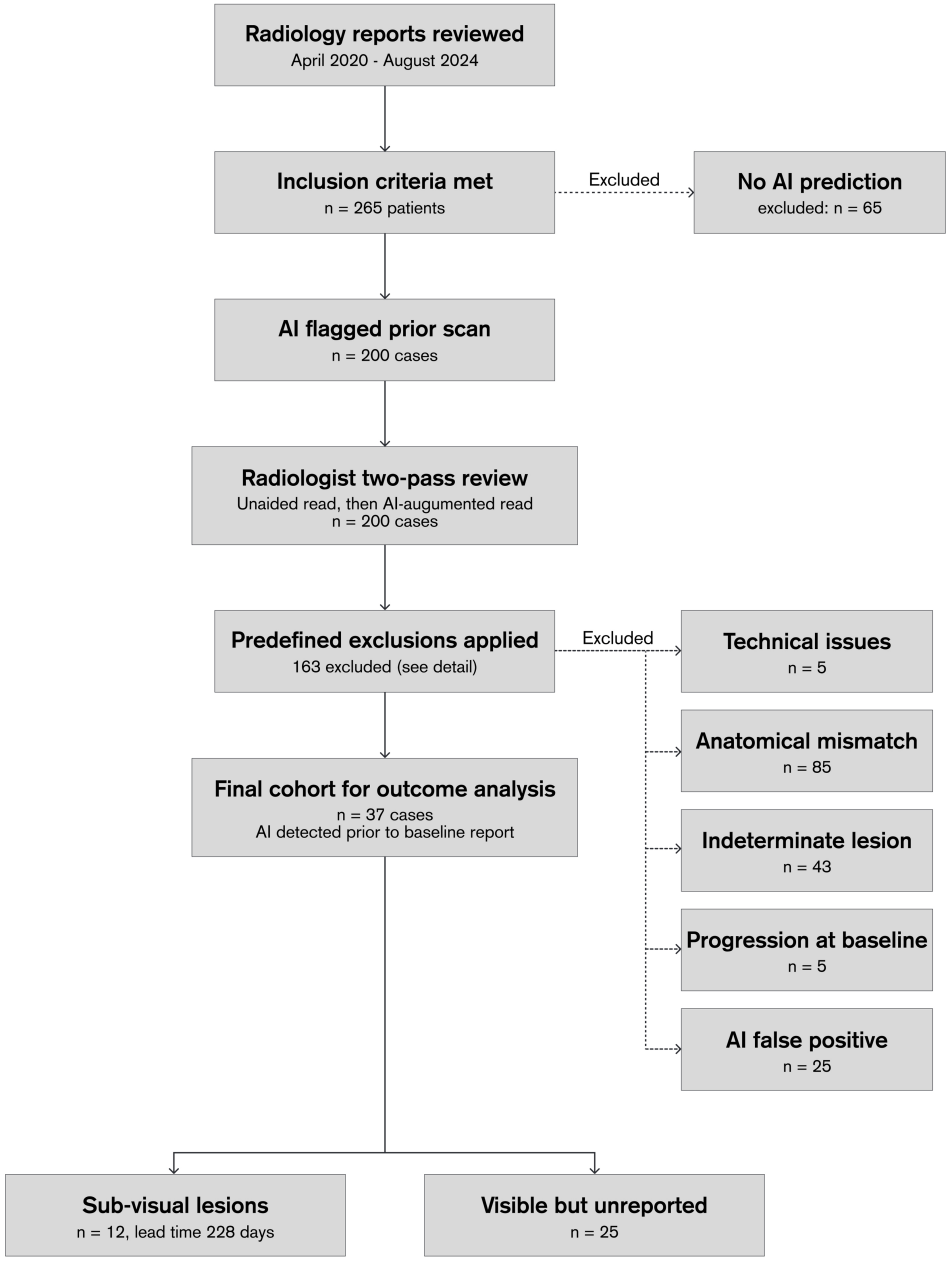
Case Selection process.

Of the 200 AI-flagged prior scans, 163 were excluded based on predefined criteria applied during the radiologist’s assessment: five were excluded due to technical or reporting issues (e.g., incorrect report linkage, missing images, invisible predictions, or patient ID mismatch); 85 were excluded because the AI-highlighted finding referred to a different anatomical location to the target malignant spinal lesion and could therefore not be used for lesion-level earlier detection assessment; 43 were excluded because the baseline lesion was indeterminate, non-malignant, diffuse, or radiographically stable, and therefore not clinically relevant for evaluating earlier detection; five were excluded because the baseline examination already showed unequivocal metastases or the report documented progression, making a prior miss implausible; and 25 were excluded because the AI finding was a false positive representing a benign or degenerative change. The remaining 37 cases were retained for outcome analysis. Figure 1 shows the selection process of cases.

### 2.3 Model description

The medical images evaluated in this study are standard-practice sagittal bone-window CT series. The spinal coverage of each series was not restricted. We therefore processed all available series, encompassing either the full spine or individual spinal segments. To handle this variability, our model operates on overlapping fixed-size 3D patches extracted from each series, with results combined via voxel-level aggregation.

We implemented a 3D Dynamic U-Net architecture for semantic segmentation of malignant spinal lesions in CT images. Semantic segmentation is an image processing technique used to classify each pixel or voxel in an image into different classes such as “malignant lesion” or “background”. In the medical field, semantic segmentation enables automated detection and delineation of organs, tissues, and lesions across modalities such as microscopy, dermoscopy, X-ray, ultrasound, computed tomography (CT), and magnetic resonance imaging (MRI). Across most applications, deep learning-based methods such as convolutional neural networks (CNNs) and encoder-decoder architectures, define the state-of-the-art for medical image segmentation tasks (Minaee et al., 2022; Seo et al., 2020; Isensee et al., 2020). The U-Net consists of an encoder-decoder structure with convolutional neural networks (CNNs), incorporating skip connections between layers of equal resolution to preserve high-resolution features (Ronneberger et al., 2015). The model outputs voxel-level masks classifying regions as background, benign lesions, or lesions with aggressive imaging features. The network was implemented using the Dynamic U-Net framework, which adapts kernel and stride sizes to the patch dimensions. Input CT scans underwent preprocessing including voxel spacing normalization, bone window intensity clipping (−500 to 1,300 HU), and intensity rescaling. Data augmentation was performed with randomized 3D spatial and intensity transformations. To process variable-sized scans efficiently, images were divided into overlapping 3D patches of fixed size and at fixed resolution. During training, patches were sampled with equal probability from lesion-containing and lesion-free regions, with 5 patches selected per image per epoch. For inference, sliding window analysis was performed with 50% overlap, and overlapping regions were aggregated using Gaussian kernel weighting (scale = 0.125). The model was trained using a weighted combination of Dice loss and cross-entropy loss and deep supervision at all three intermediate decoder layers to improve gradient flow. Stochastic gradient descent with Nesterov momentum (0.99) and L2 regularization (1e−5) was employed as the optimizer. Training utilized a learning rate scheduler with 50-epoch linear warmup followed by cosine decay, reaching a maximum learning rate of 0.01. Models were trained for 800 epochs with a batch size of 2 images. For robustness, we applied an ensemble strategy using two independent five-fold cross-validation splits, resulting in ten trained models. Predictions from all models were aggregated using a voting mechanism to generate final voxel-level segmentation maps. Post-processing included connected component analysis and anatomical filtering based on vertebral and intestinal masks obtained from a TotalSegmentator organ segmentation model (Wasserthal et al., 2023), reducing false-positive detections.

## 3 Results

From April 2020 to August 2024, 265 patients met inclusion criteria; of these, 200 had an AI prediction on the prior scan and were taken forward for radiologist evaluation. After applying predefined exclusion criteria (detailed in the Methods and Figure 1), 37 cases were identified in which the AI had flagged a malignant spinal lesion on a scan prior to the baseline report for which the AI-highlighted finding corresponded spatially to the lesion subsequently documented at baseline and confirmed as clinically relevant. The cohort comprised 113 male (56.5%), 82 female (41.0%), 2 other (1.0%), and 3 unknown (1.5%); median age 67 years (range 34–88; SD 9.11 years). Age was summarized once per patient; when multiple ages were present across exams, the per-patient median was used.

**Figure 2:**
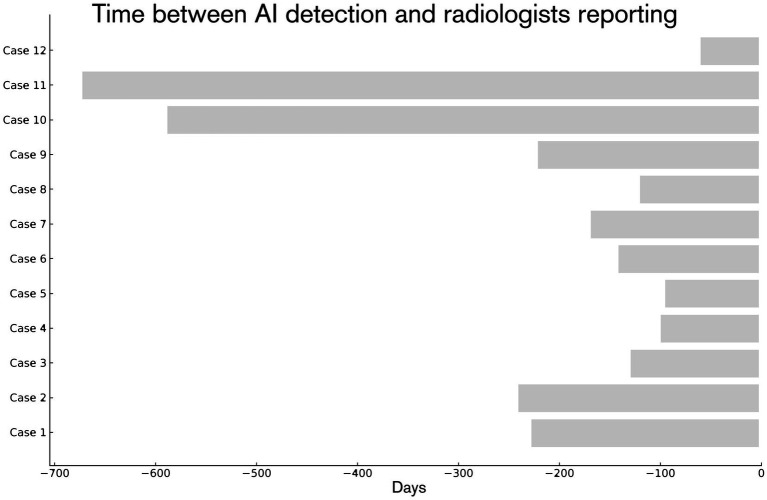
Time between AI detection and radiologist reporting.

**Figure 3:**
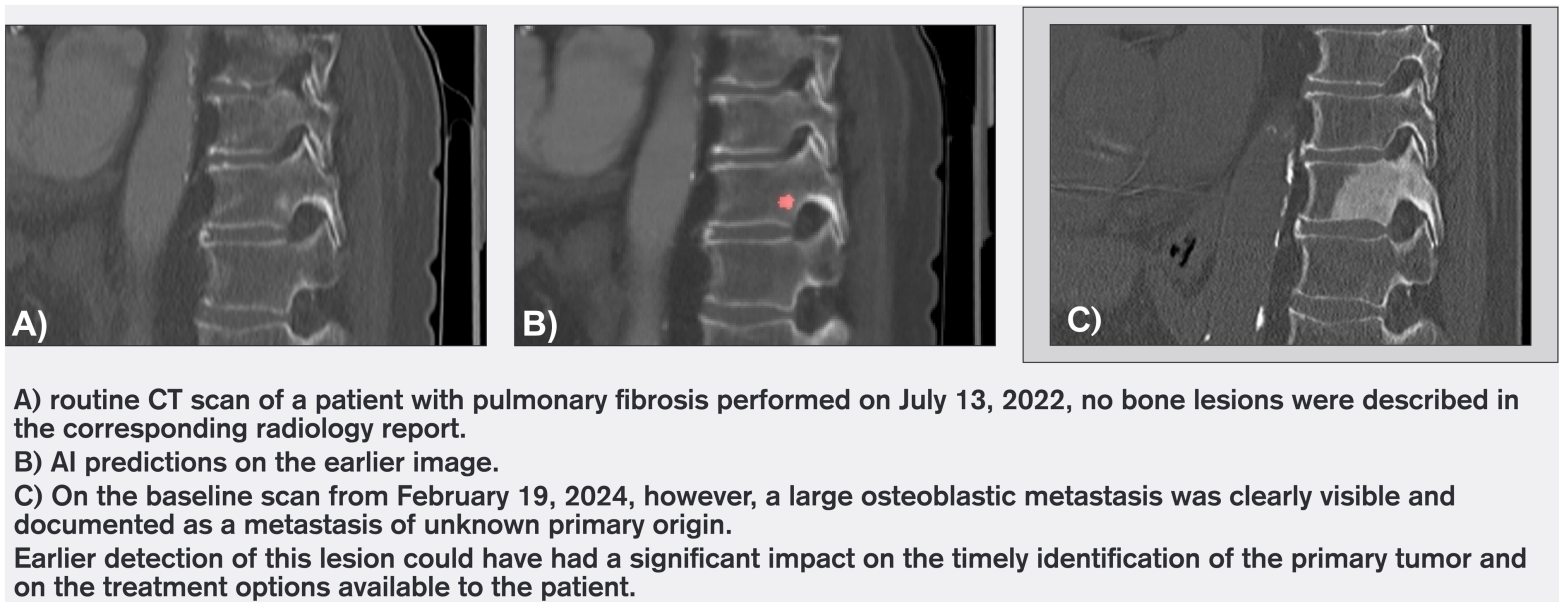
Case example 1.

**Figure 4:**
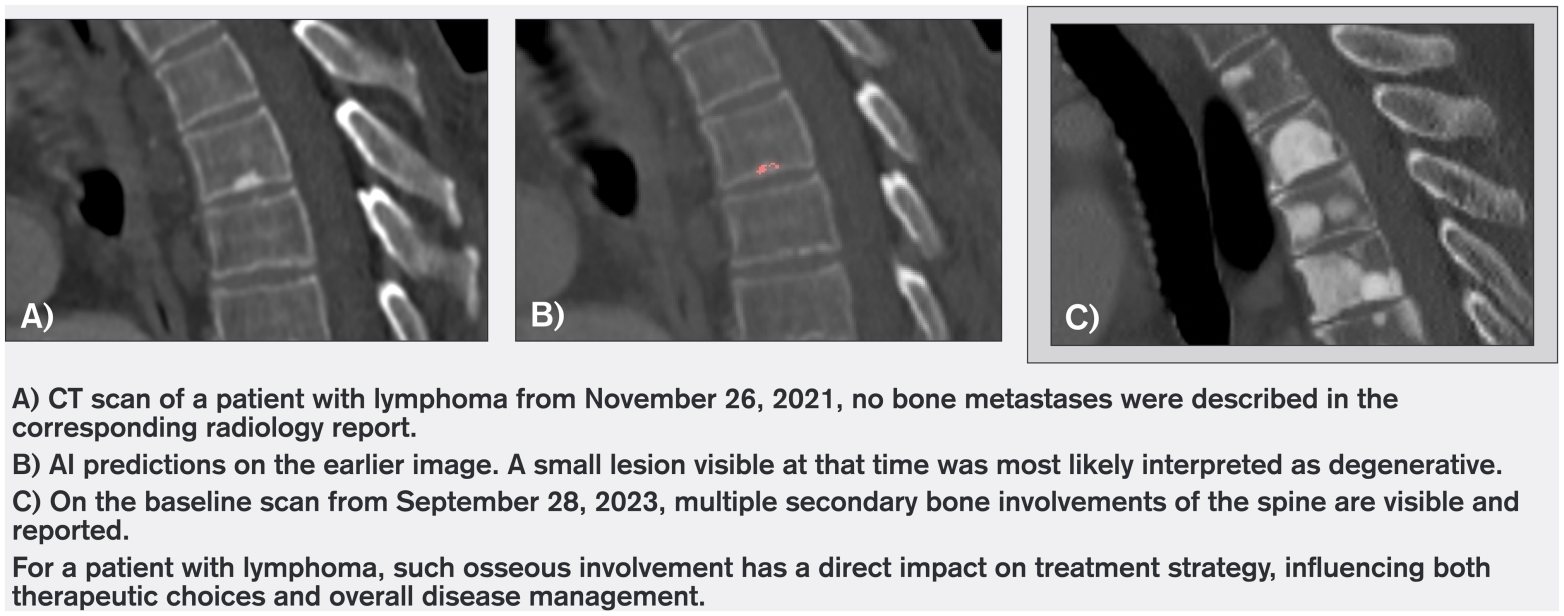
Case example 2.

Of these 37 cases, 12 lesions were not identifiable on the unaided pass and were not reported at the time of the pre-baseline scan. Their malignant nature was confirmed retrospectively, based on radiologist adjudication at baseline and subsequent disease course. Among these 12 cases, the average time between AI detection and subsequent radiologist reporting was 228 days (approximately 7.5 months; maximum 672 days). This interval was calculated as the difference between the date of the CT scan on which FloySpine CT flagged the lesion and the date of the CT scan on which the lesion was first reported by a radiologist. Figure 2 shows the time intervals, in days, between the AI-flagged prior study and the baseline study for each of the 12 cases. Because these intervals are constrained by the spacing of CT examinations, they should be interpreted as suggestive rather than definitive time-to-detection estimates. The remaining 25 lesions were retrospectively clearly visible on the pre-baseline scans and defined as malignant by the reviewing radiologist during the unaided pass, yet had not been reported in the original radiology reports at the time of the pre-baseline scan. Representative examples are shown in Figures 3, 4, illustrating cases where lesions were either barely perceptible or easily mistaken for benign findings on the pre-baseline scan but were subsequently confirmed as malignant at baseline.

## 4 Discussion

### 4.1 Principal findings

In this retrospective study, a single AI system employed as a second reader identified malignant spinal lesions on CT scans for 37 patients before they were mentioned in the initial clinical reports. Notably, 12 of these lesions were not identifiable on an unaided reading by the reviewing radiologist, suggesting that algorithmic cues may occasionally reveal sub-visual or near-threshold abnormalities. The remaining 25 lesions were visible and classified as clearly malignant upon retrospective review but had most likely been overlooked, highlighting how AI can mitigate interpretation variability related to high diagnostic workloads, limited attention, and reader experience. These factors are particularly salient in complex oncologic imaging settings. It is important to note that 163 of the 200 AI-flagged cases were excluded from outcome analysis for pre-specified reasons detailed in the Methods (see Figure 1); this high exclusion rate points to practical considerations for the deployment of lesion-level tools, and underscores that AI should serve as an assistive prompt rather than a replacement for clinical judgement, with radiologists retaining ultimate responsibility for assessing lesion relevance within the context of each patient’s individual oncologic profile.

### 4.2 Clinical implications

Using AI as a second reader can act as a safety net, increasing the likelihood that subtle findings will be identified and reviewed. This improves the completeness and internal consistency of reports, thereby strengthening clinicians’ confidence in the final interpretation. The earlier recognition of malignant spinal lesions enables more rapid initiation of systemic therapy, allows for the anticipation and prevention of skeletal-related complications (such as pathological fracture or spinal cord compression), and facilitates more effective multidisciplinary planning within individual oncologic care. Timely diagnosis and intervention in patients with spinal metastases are associated with improved functional outcomes, quality of life, and survival whereas delayed treatment is linked to poorer performance status, higher rates of neurologic deficits, and increased mortality (Lawton et al., 2019; van Tol et al., 2020). Early identification can also accelerate the initiation of systemic therapies, improving local tumor control and overall prognosis (Cao et al., 2024). Anticipating skeletal-related events, as these complications can result in irreversible neurologic deficits, pathological fractures and loss of mobility if not addressed promptly (Nakata et al., 2020). A multidisciplinary approach, incorporating oncologists, spine surgeons, radiologists and rehabilitation and pain specialists, is considered standard practice for managing malignant spinal lesions, as it enables personalized planning and timely intervention that prevent or mitigate complications (Palacio Giraldo et al., 2025). Accordingly, incorporating a reliable AI model into routine radiological reporting could minimize variability between readers and institutions, thereby ensuring consistent and comprehensive reporting across sites. Consistent with this, the medical literature indicates that AI-driven approaches in CT imaging enhance reproducibility and reporting consistency, which are critical for multicentre studies and routine clinical practice (Khosravi et al., 2025; Martin-Noguerol et al., 2023; Ong et al., 2024), and that integrating AI into radiological workflows promotes standardized, comprehensive reporting across sites. However, the literature also notes that further prospective, multicenter validation is needed to confirm generalizability and reliability in diverse clinical settings (Ong et al., 2024; Sanker et al., 2025).

### 4.3 Limitations

This study has several limitations. First, and most importantly, as a single-centre case series, this study is exploratory in nature. The sample size reflects the constraints of a proof-of-concept investigation and is not intended to yield statistically generalisable results; findings should be seen as hypothesis-generating rather than confirmatory. Second, it is retrospective, and the AI was not evaluated in a prospective, real-time clinical workflow, measured gains are therefore hypothesis-generating. Third, our ground truth relied on expert radiologist judgement at baseline and, when available, on longitudinal progression, as no additional clinical information, such as biopsy results were present in most of the cases. In 15 out of 37 cases, follow-up beyond baseline was unavailable. Although malignancy was adjudicated using anamnesis, baseline reports plus expert review, histopathological confirmation or PET-CT correlation would have strengthened the certainty further. Fourth, measured “lead times” (see Figure 2) reflect scan intervals rather than continuous monitoring. As such, intervals should be interpreted as upper-bounded proxies rather than precise time-to-diagnosis gains. Fifth, the sequential two-pass reading (first unaided, then AI-augmented) can introduce interpretive drift and recall bias. Finally, exclusions due to spatial mismatch and benign mimics emphasize that algorithm outputs must be well calibrated and tightly coupled to lesion-level correspondence if they are to inform patient-level decisions.

### 4.4 Future directions

Future work should prioritize prospective, workflow-embedded trials with clinically meaningful endpoints, such as time to treatment initiation, changes in systemic therapy choices, rates and timing of skeletal-related events, pain and functional scores, and overall survival. Study designs may include crossover or cluster-randomized reader studies to quantify improvements in sensitivity, specificity and report. Methodologically, priorities include lesion-level longitudinal tracking, harmonization across scanners/protocols, and equitable performance across demographic and disease subgroups. Where feasible, standardized reference testing (PET-CT or biopsy) should be incorporated to strengthen ground truth.

## 5 Conclusion

Our findings suggest that AI may serve as a valuable safety net for second readers in oncological CT scans of the spine. In this exploratory case study, the system identified both barely perceptible and retrospectively visible but unreported malignant spinal lesions, indicating potential to improve report accuracy and reduce variability. These results are hypothesis-generating and should not be interpreted as evidence of clinical efficacy. Prospective validation in real-time clinical workflows with rigorous outcome measures and robust safeguards against false positives will be decisive in determining whether earlier AI-flagged detection translates into better treatment decisions and improved patient outcomes.

## Data Availability

The data analyzed in this study is subject to the following licenses/restrictions: the data used in this study consist of medical imaging data provided under a data-partner agreement that permits their use only in anonymized form for internal research purposes. Due to ethical, legal, and privacy restrictions, and in accordance with the consent framework under which the data were obtained, we are not permitted to share these data publicly. The dataset includes sensitive human imaging information for which re-identification risks cannot be fully excluded; sharing would therefore breach our contractual obligations and local data-protection regulations. While the data cannot be made available upon request, all analyses were conducted using appropriately anonymized data within the secure infrastructure provided by Floy, ensuring compliance with all relevant ethical and legal requirements. Requests to access these datasets should be directed to Lea-Elena Braunschneider, braunschneider@floy.com.
